# Supplementary material for: SHREC 2025: Protein surface shape retrieval including electrostatic potential
Source: Comput Graph. Author manuscript; Available in PMC 2026 Jul 9. (PMC13344379; doi:10.1016/j.cag.2025.104394)
Supplement: 1 [file NIHMS2182104-supplement-1.pdf]

1. Post-deadline results

Table 1: **RISurProtNet results.** Submission results are results submitted before the deadline. New results were submitted after the deadline and before the release of ground truth. The main correction is the introduction of "class weighted module". We invite readers to read the discussion section to understand the reasons for the improvement.

Scores	**Submission results**	**New results**
Accuracy	0.32	0.31
Balanced Accuracy	0.09	**0.26**
F1	0.26	0.33
Precision	0.49	0.55
Recall	0.32	0.30

Table 2: **Tehrani_v2 results.** Submission results are results submitted before the deadline and before the release of ground truth. New results were submitted after the deadline and after the release of ground truth. The main correction is the use of correct transformation for each features. We invite readers to read the discussion section to understand the reasons for the improvement.

Scores	**Submission results**	**New results**
Accuracy	0.17	**0.61**
Balanced Accuracy	0.04	0.27
F1	0.15	0.57
Precision	0.22	0.59
Recall	0.17	0.61

2. Supplementary Results

Homologous Classes
34, 56, 70, 79, 80, 93
14, 31, 40, 49, 86
9, 22, 32, 47
15, 19, 39, 88
16, 76, 91
21, 64, 94
51, 53, 59
72, 82, 89
0, 12
2, 5
3, 71
4, 17
6, 11
7, 44
8, 25
10, 84
13, 66
18, 81
20, 60
23, 83
26, 62
28, 92
29, 33

Homologous Classes
35, 95
36, 38
37,77
41, 43
45, 58
46, 96
48, 78
50, 74
54, 57
61, 90
63, 75
65, 87
67, 68
73, 85
1
24
27
30
42
52
55
69

Figure 1: List of Homologous Classes in each row. Two homologous classes share 90-98% sequence similarity. Due to this similarity, the folding and the shape can be similar between two homologous proteins.

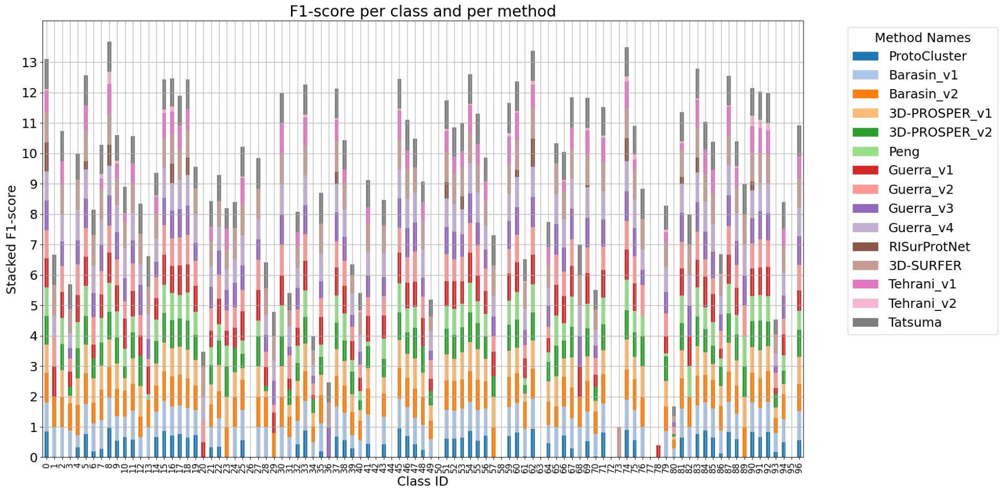

(a) Stacked-F1 per class

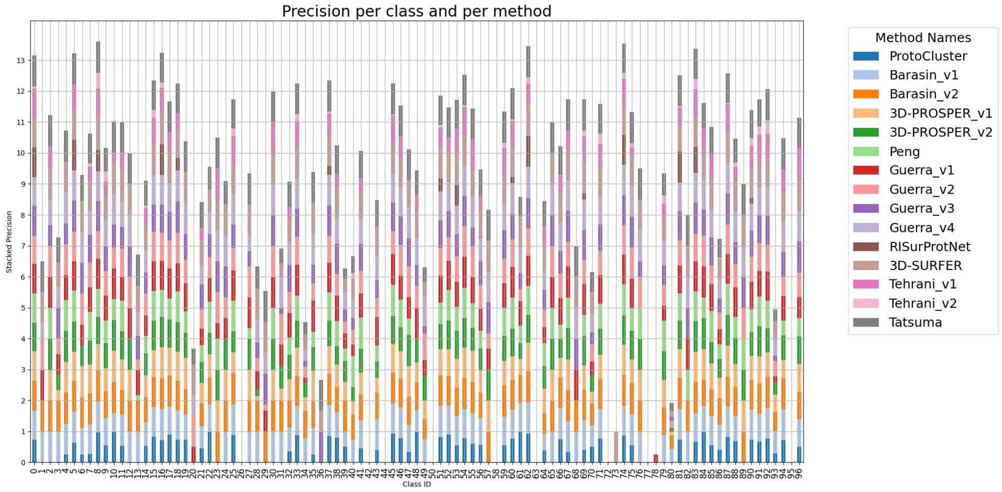

(b) Stacked-Precision per class

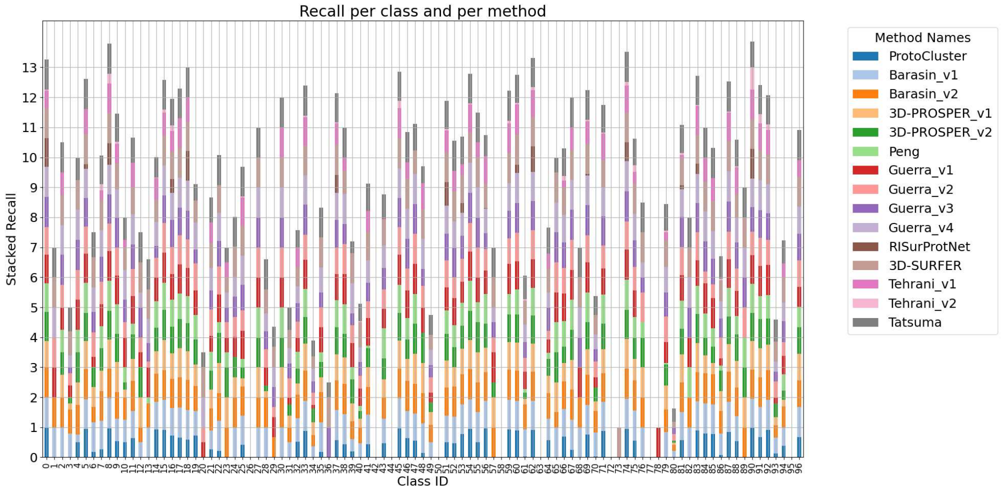

(c) Stacked-Recall per class

Figure 2: **Stacked-F1, stacked-Precision and stacked-Recall.** The "stacked-metric" (metric = F1, Precision or Recall) is the sum of the considered metric of all methods per class. X-axis corresponds to Class ID, and Y-axis corresponds to the sum of scores, between 0 and 15.

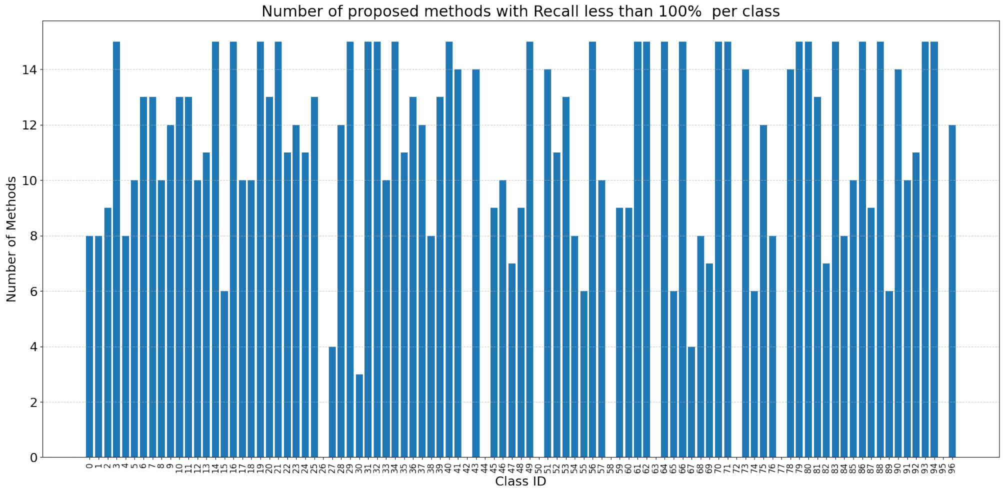

(a) Number of methods with Recall less than 100% per class

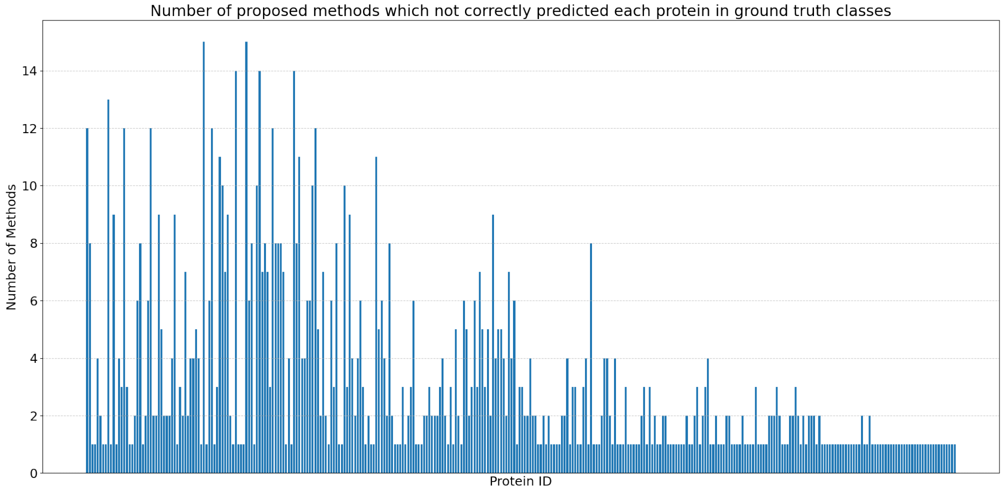

(b) Number of methods which not correctly predicted each protein in ground truth class

Figure 3: [A] Count of the number of proposed methods where Recall was less than 100% per class. For instance, we observe 25 classes {3, 14, 16, 19, 21, 29, 31, 32, 34, 40, 49, 56, 61, 62, 64, 66, 70, 71, 79, 80, 83, 86, 88, 93, 94} where all methods have not correctly predicted at least one protein surface in these ground truth classes. [B] Count the number of proposed methods which not correctly predicted each protein in ground truth classes. For instance, two proteins were not correctly predicted in ground truth classes by all methods.

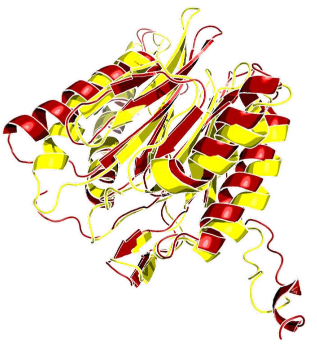

(a) Structural Fold between two conformers

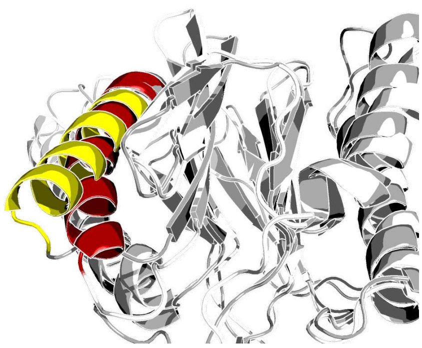

(b) Focus on local deviation

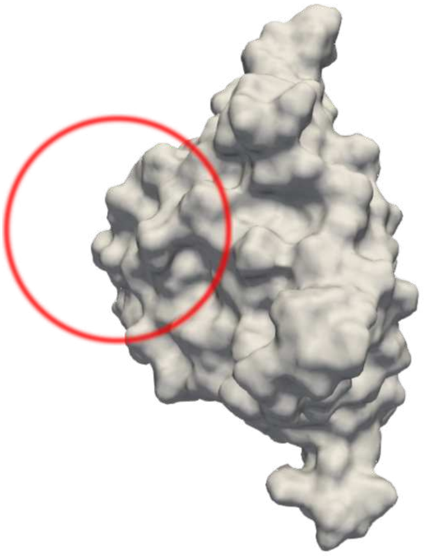

(c) Surface representation: 6epc_14:N:7

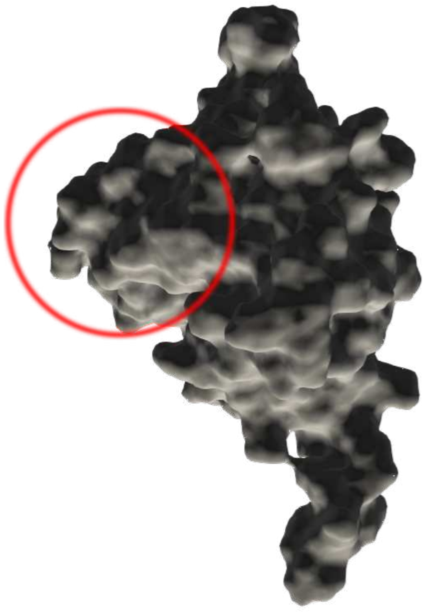

(d) Surface representation: 3unb_13:AA:3

Figure 4: Structural and surface representation of two conformers in class 29. (A) Structural superposition between two conformers with a global similar folding, (B) shows the structural alignment between 6epc and 3unb. Although the global fold can be similar, there is local deviations as illustrated by α-helix. This variation leads to change in local shape (C, D). Because the training set is composed only of shapes identical to 3unb, 6epc is misclassified by all methods in non-homologous classes.

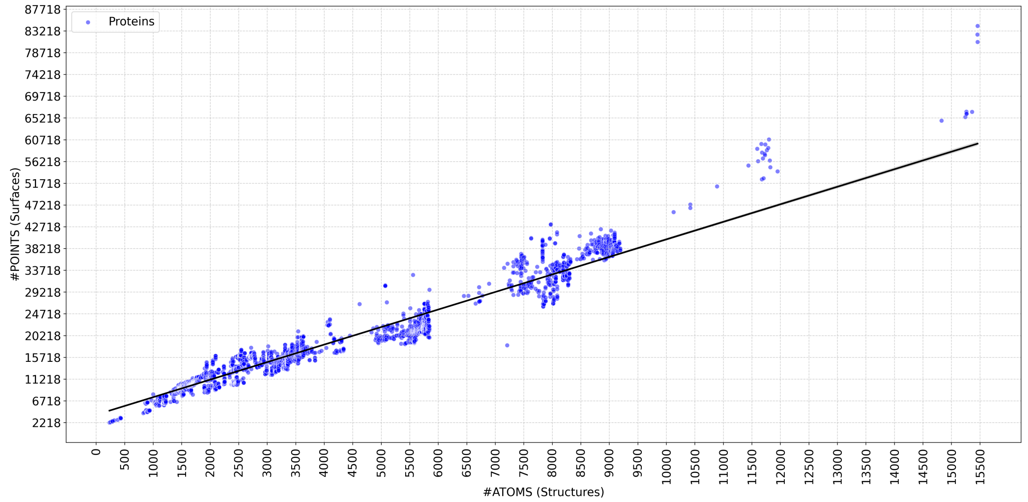

Figure 5: Correlation between the atom numbers from structures and point numbers from surfaces for each protein from the dataset. The number of atoms is to the number from PDB files of each protein. The number of points is the number from VTK files. The number of points is between 2,000 and 90,000 points. There is a correlation between the number of atoms and the number of points.

3. Method specifications: hardwares, epochs and runtimes

*3.1. **By:** A. Tatsuma*

Statistics of local features and potentials.

All processes in our method were executed on the following computer environment: Intel Core i3-14100 3.5GHz (4 physical cores, 8 threads via Hyper-Threading), 16GB RAM, Ubuntu 24.04.2 LTS on WSL2 of Windows 11. Computations were performed solely on the CPU, without utilizing a GPU.

The average execution time for feature extraction was 1.55 seconds per model, for SVM training was 2.95 seconds, for predicting class was 2.81 milliseconds. The total runtime for feature extraction on all models, SVM training, and class prediction on all test data was 4 hours 12 minutes 58 seconds.

*3.2. **By:** T. Barisin, E. Rusakov, U. Göbel*

Simplified RIConv++: Rotation Invariant Deep Network for Point Clouds.

Model is implemented in python using pytorch library. All models were trained for 250 epochs using Adam optimizer with a default learning rate of 0.001 and step learning rate decay every 20 epochs on a single GPU NVIDIA RTX 3500 Ada with Intel i7 - 13700HX CPU. The training set was split into 80:20 and used the last 20% of data for validation. For conversion from vtk to point cloud of size 8,196 points, only CPU and trimesh library were used. Point cloud was normalized to fit into unit sphere, while chemical features was normalized to be in [0,1] interval for every mesh separately.

For both Run 1 and Run 2, point cloud extraction took 6 hours 20 minutes and 1 hour 30 minutes for train and test, respectively. Training took 23 hours for Run 1 and 28 hours for Run

2, testing took 255 seconds for Run 1 and 195 seconds for Run 2.

*3.3. **By:** Y. Peng, S. Deng*

3D protein recognition based on 2D images.

Training and evaluation were conducted on workstations equipped with the 13th generation Intel Core i7-13700F CPU (16 cores, 24 threads) and NVIDIA GeForce RTX 3070 Ti (8GB) GPU. This code was implemented in Python 3.9.21 using PyTorch, PyTorch Geometric (PyG), and Scikit learn. The entire training process spanned 30 cycles, with a training time of approximately 3,931 seconds. Evaluating the test set took about 11 seconds and extracting high-dimensional features took 50 seconds.

*3.4. **By:** Y. Kagaya, J.H. Park, D. Kihara*

3D-SURFER: Protein surface classification using 3D Zernike descriptors (3DZD).

Our method required only CPUs. All predictions were generated on a workstation equipped with a single AMD EPYC 7443P processor (24 cores, 48 threads).

The preprocessing steps, calculating 3DZDs from given 11,565 surfaces (9,244 training and 2,321 testing), required an average of 9 seconds per surface using a single thread. This step was parallelized across 32 threads, resulting in a total wall time of approximately 3,200 seconds for the entire test set. To identify the most similar surface, all-to-all Euclidian distances were computed between the 2,321 samples in the test dataset and the 9,244 samples in the training dataset using the 'cdist' function from the SciPy library. This distance computation took approximately 4 seconds in total.

*3.5. **By:** M. Guerra, G. Palmieri, A. Ranieri, U. Fugacci, S. Biasotti*

Topological descriptors (Guerra_v1).

For hardware, a processor Intel(R) Core(TM) i9-9900KF CPU @ 3.60GHz, 64bits, memory of 32GiB and GPU NVIDIA Corporation TU117 [GeForce GTX 1650] were used, with CUDA enabled.

Computation of **descriptors** for training and test set took around 3 hours in total. Training of the **classifier** took around 30 minutes for 2,000 epochs.

PointNet architecture (Guerra_v2).

For the final evaluation, the model was trained on the full training set for 100 epochs. Experiments were conducted on a workstation equipped with three *NVIDIA RTX A6000 GPUs* (48GB VRAM each), an *AMD Ryzen Threadripper PRO 7965WX CPU* (24 cores/48 threads), and 128GB DDR5 RAM. The training used just one of the three GPUs.

A multi-view image-based approach using a ViT encoder (Guerra_v3).

Experiments were conducted on a workstation equipped with three *NVIDIA RTX A6000 GPUs* (48GB VRAM each), an *AMD Ryzen Threadripper PRO 7965WX CPU* (24 cores/48 threads), and 128GB DDR5 RAM. Training utilized two GPUs, completing 10 epochs in 20 hours (~ 2 hours/epoch). Inference times averaged 2.5 seconds per protein screenshot generation (test set) and 150 milliseconds per forward pass using the trained model.

3.6. *By: R. He, H. Benhabiles, A. Cabani, K. Hammoudi*

3D-PROSPER: 3D Protein Representation via Optimized Self-supervised and multi-task Point cloud Encoding for Recognition.

This framework has been implemented in Google Colab, Colab GPU runtime comes with an Intel Xeon CPU @2.20 GHz, 13 GB RAM, a Tesla K80 accelerator, and 12 GB GDDR5 VRAM. The running times obtained on the test set using two inference models were as follows for one protein: 0.242 seconds for data pre-processing (step 0) and 0.082 seconds for inference models (step 2 or step 3).

3.7. *By: H. Li, H. Huang, C. Li*

RISurProtNet.

Three NVIDIA RTX 3090 GPUs (total 76 GB VRAM) and CPU AMD EPYC 7542 (64 cores) were used. Each epoch took approximately 15 minutes, and trained for 55 epochs (about 1/9 of the 450 epochs used in the original paper) in total (around 14 hours of training time).

3.8. *By: A. Tehrani, F. Meng, F. Heidar-Zadeh*

Siamese Deep Metric Learning on Property-Spectrum (Tehrani_v1).

The entire pipeline was implemented in Python 3.10 using PyTorch and scikit-learn. Siamese network had one hidden layer producing 480-dimensional embeddings, applies 0.8 dropout, and was trained for 50 epochs with Adam (learning rate set to 0.001) and a triplet loss function (margin set to 25). The downstream MLP classifier used three layers (512, 256, 126), 0.83 dropout, and was trained for 600 epochs with AdamW (learning rate set to 0.01). Experiments ran on an NVIDIA Quadro GP100 GPU and an Intel Xeon Gold 6130 CPU (16 cores and 32 threads) with 12 GB RAM. Computing 15 property spectra required 126 seconds, and aggregating all 15 property-spectra took 25 minutes using 15 parallel CPU cores. Training the full pipeline completed in under 15 minutes, and evaluation on the test set completed in under 2 minutes.

Gradient Boosting LightGBM Model on Property-Spectrum (Tehrani_v2).

Data was stratified into 90% training and 10% test, then trained the model using LightGBM and optuna (Python 3.11.5), which took 40 minutes to complete. Predicting the test data took less than a minute. All model training was performed using a single Intel(R) Xeon(R) Gold 5118 CPU (24 processors) provided by the Center for Advanced Computing at Queen's University.

3.9. *By: T.A. Yang*

ProtoCluster.

Experiments were carried out using Google Colab's free CPU and GPU services, with an Intel Xeon CPU running at around 2.2–2.3 GHz34, 1 processor, 1 core, and 2 threads, T4 GPU (NVIDIA Tesla T4) 2,560 CUDA cores, 16 GB GDDR6 memory.

Concerning runtime, it took on average 20.31 seconds per mesh for preprocessing (CPU), for test-set evaluation, it took 1 hour 18 minutes 52 seconds for feature extraction (CPU) and 16 seconds for model inference (GPU), and it took 25 minutes for every 20-epoch training session, with a 3-stage training method that sums up to 75 minutes.
